# A spicy twist: A rare case of anaphylaxis to banana pepper

**DOI:** 10.1016/j.jacig.2025.100496

**Published:** 2025-05-17

**Authors:** Ishitha Jagadish, Phoebe Newell, Ricardo J. Estrada-Mendizabal, Saiyara S. Shama, Alexei Gonzalez-Estrada

**Affiliations:** a Department of Internal Medicine, University of Arizona Phoenix, Phoenix, Ariz; b Division of Allergy, Asthma, and Clinical Immunology, Department of Internal Medicine, Mayo Clinic, Scottsdale, Ariz

**Keywords:** Anaphylaxis, food allergy, *Capsicum*, *Capsicum annuum*, banana pepper, pepperoncini

## Abstract

This case report describes the first documented instance of banana pepper (*Capsicum annuum*)-induced anaphylaxis in a 30-year-old male with intermittent asthma and allergic rhinitis, confirmed through positive prick-to-prick testing.

Anaphylaxis is a severe, potentially life-threatening allergic reaction that can be triggered by various foods, medications, and environmental exposures. Although food-induced anaphylaxis is often attributed to common allergens such as peanuts, shellfish, and tree nuts, less common triggers can be easily overlooked. *Capsicum* cultivars, which include bell peppers, chili peppers, banana peppers, and more, are rare but potential causes of anaphylaxis. Reports of anaphylaxis in response to banana peppers (*Capsicum annuum*) are particularly sparse, making it a less-recognized allergen with unknown incidence or prevalence. This case report presents a rare instance of anaphylaxis in a 30-year-old male with asthma and allergic rhinitis following the consumption of a spiced lamb dish containing banana peppers. Given the exacerbation of asthma symptoms after anaphylaxis in this patient, the report further discusses the need for reassessment of asthma control and personalized patient education.

A 30-year-old male with asthma and allergic rhinitis had been effectively managing both conditions with as-needed albuterol and over-the-counter antihistamines without any recent changes to his medication regimen. Incidentally, he developed wheezing, dyspnea, hoarseness, pruritic lips without angioedema or urticaria, and abdominal pain within 10 minutes of consuming a spiced lamb takeout dish. At time of presentation to the emergency department, his vital signs were normal. He was treated with 1 dose of epinephrine, intravenous steroids, and histamine-1 and histamine-2 receptor blockers, leading to complete resolution of his symptoms within 3 hours. An allergist confirmed the contents of the dish with the restaurant owner. It included lamb, salt, ground red pepper, black pepper, onions, basmati rice, tomato, pita, basil, butter, and pepperoncini (a type of banana pepper). The patient had no history of anaphylaxis to food allergens and could not recall previous consumption of this specific variety of banana pepper. He actively avoided lamb and banana pepper ingredients following the index reaction. Skin and prick-to-prick testing revealed a negative result for fresh lamb but a positive result for banana pepper (Fig 1), confirming the diagnosis of anaphylaxis in response to the pepper. Additionally, banana pepper prick-to-prick testing was performed on 8 healthy controls, with 7 of those tested having negative results. Serum-specific *C annuum* IgE was not commercially available at our institution. The patient was advised to avoid all *Capsicum* cultivars and instructed on use of an epinephrine autoinjector. His asthma, which had previously been controlled with as-needed albuterol, was exacerbated by the allergic reaction, resulting in daily dyspnea for 1 week. He reported no asthma-related nighttime awakenings in the preceding month, nor did he have any acute asthma exacerbations or need for systemic steroids in the previous year. Given the likely association between the patient’s allergic reaction and now-uncontrolled intermittent asthma, a baseline serum tryptase level was ordered and determined to be 6.8 ng/mL. The patient was counseled on proper albuterol inhaler use. As of 6 months after the incident, the patient has been successfully avoiding consumption of or contact with *Capsicum* cultivars.

**Fig 1:**
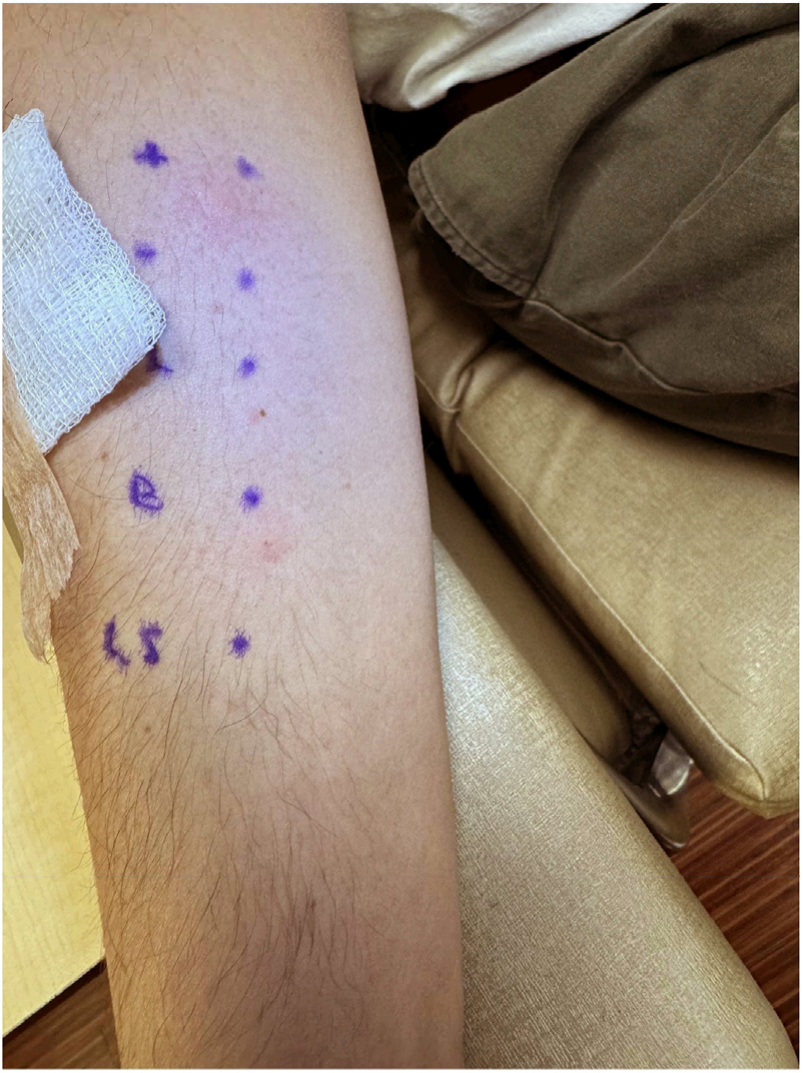
Prick-to-prick testing results. The results of prick-to-prick testing were negative (wheal size, 0 mm; flare size, 0 mm flare) for fresh lamb (L) and positive (wheal size, 5 × 8 mm; flare size, 8 ×10 mm) for banana pepper (B).

Although *Capsicum* cultivars are widely consumed, reports of anaphylaxis in response to them are rare. A PubMed search using the key term *Capsicum anaphylaxis* yielded only 8 articles published since 1998, most of which were case reports involving bell pepper, pickled chili pepper, paprika, or *Capsicum* spray.^1^, ^2^, ^3^, ^4^, ^5^, ^6^, ^7^, ^8^ The remaining 2 studies analyzed sensitization profiles in response to bell pepper and immunoreactive proteins in *Capsicum*-based spices.^7^^,^^8^ To the best of our knowledge, this case report presents the first documented instance of anaphylaxis triggered by banana pepper, contributing to the limited literature on *Capsicum*-induced allergic reactions.

The identification of banana pepper as the allergen in this case was achieved through a meticulous review of the patient's dietary history and verification of culprit dish’s ingredients with the restaurant owner. Such investigations are crucial for achieving accurate diagnosis and identification of possible trigger(s). Patient education on avoidance of *Capsicum* cultivars and proper use of epinephrine autoinjectors was essential for future prevention. If the patient wished to reintroduce *Capsicum* plants into his diet, an expanded skin prick testing panel could be considered. However, not much is known about potential cross-reactivity among different *Capsicum* species; hence, desensitization strategies have not been officially established.^8^ Additionally, the post-anaphylactic exacerbation of asthma symptoms in this patient emphasizes the need for reassessment of asthma control and counseling on the appropriate use of rescue medications.

The scarcity of literature regarding *Capsicum*-induced allergic reactions underscores the need for awareness among health care providers about the potential of less common food allergens to provoke severe reactions in sensitized individuals. More extensive epidemiologic studies are warranted to determine the prevalence and incidence of anaphylaxis to *Capsicum* species. Given the presumed rarity of such occurrences, future research should focus on identifying and characterizing the allergic proteins within various *Capsicum* plants to help investigate cross-reactivity, diagnostic strategies, and management options such as desensitization and/or allergen immunotherapy. Because of the potential variability of allergens among *Capsicum* cultivars, there may be a need to develop expanded standardized testing panels for skin prick testing and specific IgE testing to improve diagnostic accuracy. Following anaphylactic reactions, allergists should assess and manage any coexacerbations of chronic conditions, such as asthma, to ensure comprehensive care. Patients should be advised on how to avoid triggers, when and how to use epinephrine autoinjectors, and when to seek urgent evaluation. Long-term follow-up is crucial to evaluate the effectiveness of avoidance strategies and their impact on the patient’s quality of life.

## Disclosure statement

Disclosure of potential conflict of interest: The authors declare that they have no relevant conflicts of interest.
